# A series of pyrimidine-based antifungals with anti-mold activity disrupt ER function in *Aspergillus fumigatus*

**DOI:** 10.1128/spectrum.01045-24

**Published:** 2024-06-25

**Authors:** Martin T. Kelty, Aracely Miron-Ocampo, Sarah R. Beattie

**Affiliations:** 1 Department of Pediatrics, Carver College of Medicine, University of Iowa, Iowa City, Iowa, USA; Universidade de Sao Paulo, Sao Paulo, Brazil

**Keywords:** antifungals, *Aspergillus fumigatus*, mold, high-throughput screening

## Abstract

Fungal infections are a major contributor to morbidity and mortality among immunocompromised populations. Moreover, fungal disease caused by molds are difficult to treat and are associated with particularly high mortality. To address the need for new mold-active antifungal drugs, we performed a high-throughput screen with *Aspergillus fumigatus*, the most common pathogenic mold. We identified a novel, pyrimidine-based chemical scaffold with broad-spectrum antifungal activity including activity against several difficult-to-treat molds. A chemical genetics screen of *Saccharomyces cerevisiae* suggested that this compound may target the endoplasmic reticulum (ER) and perturb ER function and/or homeostasis. Consistent with this model, this compound induces the unfolded protein response and inhibits secretion of *A. fumigatus* collagenases. Initial cytotoxicity and pharmacokinetic studies show favorable features including limited mammalian cell toxicity and bioavailability *in vivo*. Together, these data support the further medicinal chemistry and pre-clinical development of this pyrimidine scaffold toward more effective treatments for life-threatening invasive mold infections.

**IMPORTANCE:**

Invasive fungal diseases are life-threatening infections caused by fungi in immunocompromised individuals. Currently, there are only three major classes of antifungal drugs available to treat fungal infections; however, these options are becoming even more limited with the global emergence of antifungal drug resistance. To address the need for new antifungal therapies, we performed a screen of chemical compounds and identified a novel molecule with antifungal activity. Initial characterization of this compound shows drug-like features and broad-spectrum activity against medically important fungi. Together, our results support the continued development of this compound as a potential future therapy for these devastating fungal infections.

## INTRODUCTION

Molds are filamentous, environmental fungi that cause invasive disease in immunocompromised individuals. Patients at risk of invasive mold infections (IMIs) include those undergoing chemotherapy, long-term steroid treatment for transplant or graft-versus-host disease, and specific immunomodulatory therapies. Currently, there are three classes of antifungals used to treat invasive fungal disease: azoles (e.g., voriconazole), echinocandins (e.g., caspofungin), polyenes (e.g., amphotericin B), and the pyrimidine-based anti-metabolite, 5-flucytosine, which is used only in combination therapy to treat cryptococcosis, due to the rapid emergence of antifungal resistance when used as monotherapy (1). However, a major deficit in these clinical antifungals is broad-spectrum activity against molds (2). The introduction of the second-generation azoles in the early 2000s, including voriconazole and posaconazole, greatly improved the mold activity of this class of drugs (3), particularly against the most common pathogenic mold isolated from IMIs, *Aspergillus fumigatus*. The second-generation azoles improved the survival rates of invasive aspergillosis to ~30% to 50% (4, 5), though mortality of disseminated disease remains high, reaching 80%–100% when *A. fumigatus* disseminates to the brain (4, 6). The introduction of yeast- and *Aspergillus*-active antifungal prophylaxis in susceptible patient populations has reduced the overall number of invasive fungal disease in these patients, but it has come at the cost of an increase in breakthrough mold infections. These breakthrough infections have increased the proportion of IMIs caused by non*-fumigatus Aspergillus* spp. and by historically “rare” molds such as *Fusarium*, *Mucor*/*Rhizopus*, and *Scedosporium*/*Lomentospora* spp. (7, 8). Though these molds are still much less common than *A. fumigatus* clinically, management of these infections remains a significant problem. Among these genera, intrinsic antifungal resistance to some, if not all, clinical antifungals results in poor clinical response and contributes to extremely high mortality rates of invasive disease, reaching 80%–100% (9, 10). Though these second-generation azoles also improved the activity against *Mucor*/*Rhizopus* spp., their activity is still variable against the rare molds, particularly *Scedosporium* and *Fusarium* spp., which are sometimes referred to as “untreatable.” Thus, there is a critical need for novel antifungals with potent broad-spectrum activity against molds.

IMIs are among the most aggressive form of invasive fungal disease due to difficulty in diagnosis and lack of efficacious treatment (11, 12), and the organism’s ability to cause tissue destruction. As environmental saprophytes, molds obtain many of their nutrients through the degradation of complex substrates by secretion of an array of enzymes into their environment. The host environment is no exception, where the secretion of enzymes is linked to host damage, tissue destruction, and angioinvasion (13–15). Secretion affords fungi the ability to degrade and consume nutrients, localize critical adhesion and host interacting proteins to the fungal cell surface, and induce tissue damage. However, this highly active secretory machinery results in the accumulation of unfolded or misfolded proteins, which generates stress and perturbs normal endoplasmic reticulum (ER) function. Thus, pathways such as the unfolded protein response (UPR), which responds to perturbations in ER homeostasis, are essential for fungal virulence (16). As a critical aspect of fungal pathogenesis, disruption of ER homeostasis is being explored as a strategy to kill fungi within the host while potentially reducing associated tissue destruction (17).

To address the need for mold-active antifungals, we previously developed a high-throughput, luciferase-based screening assay to screen directly with *A. fumigatus* (18). This assay measures fungal cell lysis through detection of the cytosolic enzyme adenylate kinase (AK) released into the supernatant. AK converts ADP to ATP which generates light in the presence of AK detection reagent (19). A unique feature of this assay when used with *A. fumigatus* is the ability to also detect compounds that inhibit germination. During vegetative growth, *A. fumigatus* releases AK, generating background signal which is diminished when conidia fail to germinate. This allows us to capture two separate readouts of antifungal activity in one screen (18). With this assay, we have identified candidate mold-active antifungals including a novel family of ketoalkylpyridinium compounds that are under investigation for use as topical treatments for fungal keratitis, surface disinfection, and catheter-associated fungal infections (20). These compounds are not suitable for systemic use for invasive disease due to toxicity. Thus, we have expanded our screening efforts to include a library of chemically diverse, synthetic drug-like molecules.

Here, we apply this screening strategy to a library of ~50,000 small molecules and identify a novel, pyrimidine-based scaffold with broad-spectrum anti-mold activity. Using a yeast chemically-induced haploinsufficiency screen, we identified the mode of action as disruption of ER homeostasis, which results in reduced secretion of collagenase enzymes in *A. fumigatus*. Though the specific molecular target and mechanism of action of this compound is unknown, we demonstrate a structure-activity relationship among a series of structural analogs, minimal mammalian cell toxicity, and favorable initial *in vivo* pharmacokinetics. These results support the continued development of this scaffold to identify a lead compound to treat life-threatening IMIs.

## RESULTS

### A novel, broad-spectrum pyrimidine-based antifungal small molecule

To identify novel anti-mold small molecules, we performed a screen of ~50,000 synthetic, drug-like compounds against *A. fumigatus* using the AK-based screening platform to detect the inhibition of fungal germination and lysis of *A. fumigatus* hyphae (18). Our primary screen yielded 227 hits, which were subject to secondary screening to remove noise, inhibitors of AK, and inhibitors of luciferase. Following secondary screening with a resazurin-based assay, we identified 44 validated hits that either increased AK signal by six median absolute deviations (MADs) from the plate average (fungilytic compounds) or those where AK signal was decreased by 3.5 MADs and inhibited *A. fumigatus* growth by at least 60% as determined by resazurin (inhibitors of growth/germination).

**Fig 1:**
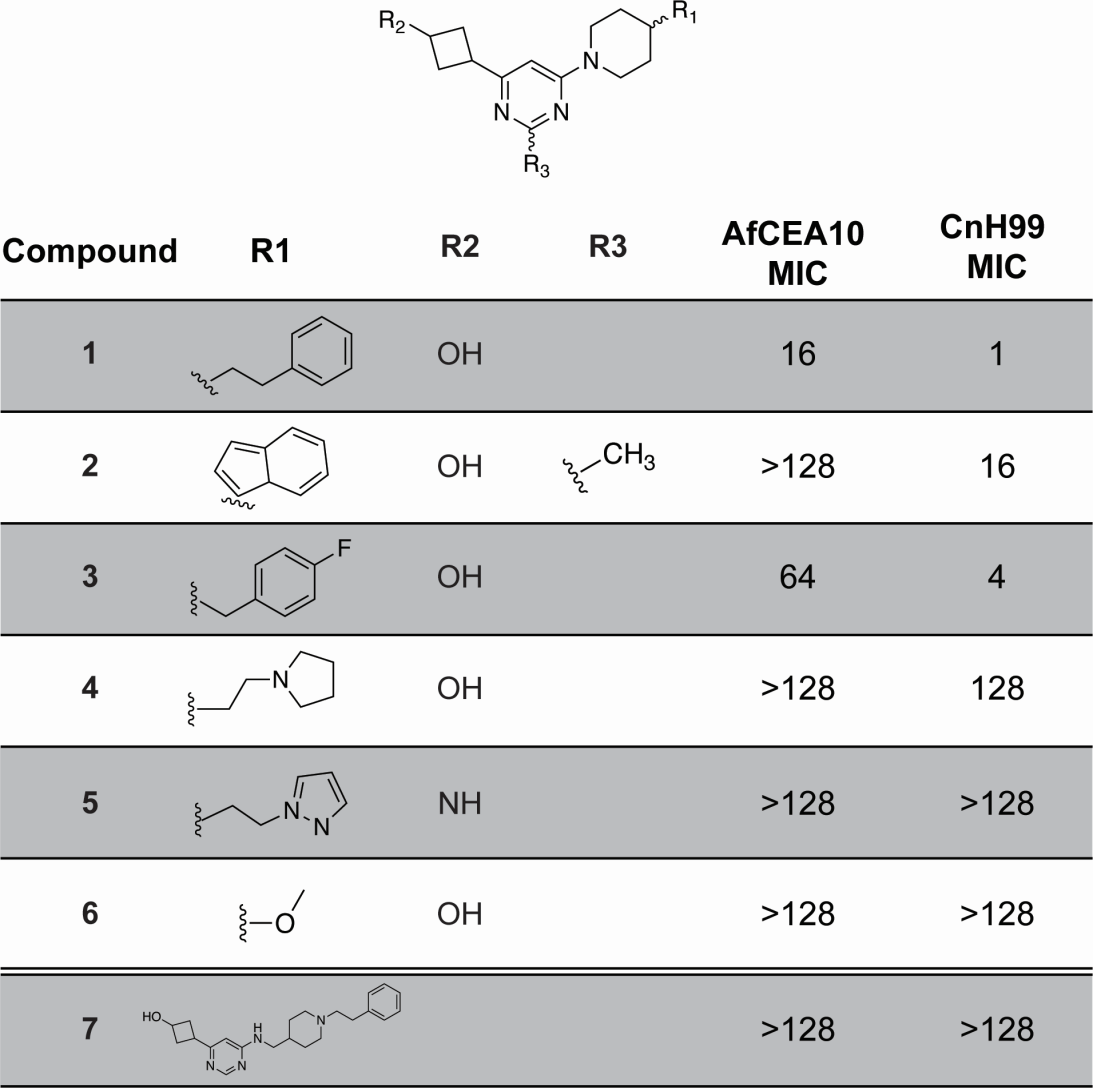
Structure-activity relationship of pyrimidine-based antifungals. Pyrimidine-based scaffold labeled with R_1_, R_2_, and R_3_ groups. For each analog, substitutions of each R group are shown along with *A. fumigatus* (Af) CEA10 and *C. neoformans* (Cn) H99 MICs (µg/mL). For 7, full structure is shown.

Among the validated hits that inhibited growth/germination, we identified a novel series of pyrimidine-based molecules, including compound **1** (Fig. 1). The minimum inhibitory concentration (MIC) of **1** is 8–16 µg/mL against *A. fumigatus* reference strains and clinical isolates. Further testing showed that it has similar activity against less common pathogenic molds including *Aspergillus terreus*, *Aspergillus niger*, *Mucor circinelloides*, and *Lomentospora prolificans* (Table 1). Strikingly, we saw potent activity against a lab reference strain of *Scedosporium apiospermum* with an MIC of 2 µg/mL. We also observed activity against pathogenic yeast species including *Cryptococcus neoformans* (2 µg/mL), moderate activity against *Candida albicans* (64 µg/mL), and activity against one of two tested *Candida auris* strains (3,081; MIC = 8 µg/mL; Table 1).

**TABLE 1:** MICs of **1** against medically important yeast and mold species^*a*^

Species	Strain	MIC
*A. fumigatus*	CEA10	16
	AF293	16
	ATCC13073	8
	SPF98^*d*^	8
	SPF93	8
	F15861^*c*^^,^^*d*^	8–16
	F16867^*c*^^,^^*d*^	8–16
	S001^*c*^	8
	S003^*c*^	4
*A. terreus*	S010^*c*^	8
	S023^*c*^	16^*b*^
*A. niger*	S007^*c*^	16^*b*^
*M. circinelloides*	CBS277.49	16
*L. prolificans*	90853	8
*S. apiospermum*	MYA3635	2
*F. oxysporum*	DUMC Fo^*c*^	>64
*C. neoformans*	H99	1
	JEC21	2
	DUMC111^*c*^	1
	DUMC118^*c*^	2
	DUMC138^*c*^	1
*C. albicans*	SC5314	64
	TWO7241^*c*^^,^^*d*^	64
	TWO7243^*c*^^,^^*d*^	64
*C. glabrata*	KK2001	64
*C. auris*	0390	>128
	3081^*c*^^,^^*d*^	8
*S. cerevisiae*	BY4743^*c*^	8

^*a*^ Minimum inhibitory concentrations (MICs, µg/mL) under Clinical and Laboratory Standards Institute conditions, representative of two independent experiments.

^*b*^ Minimum effective concentration (MEC; no clear well, growth reduced by ≥50% at MEC).

^*c*^ Clinical isolate.

^*d*^ Drug-resistant isolate.

Importantly, we tested drug-resistant *A. fumigatus* and *C. albicans* isolates with a variety of defined mechanisms of resistance and did not observe changes in MIC (Table 1). The azole-resistant *Candida* strains (TWO7241 and TWO7243) (21) are clinical isolates with increased expression of the ABC transporter, Cdr1, and the major facilitator family pump, Mdr1, efflux pumps commonly associated with multidrug resistance in *C. albicans***.** These results indicate that **1** is not subject to drug efflux by these commonly upregulated pumps in azole-resistant *C. albicans* isolates. In addition, we tested *A. fumigatus* voriconazole-resistant strains with mutations in the azole target, Cyp51. The most common mutation isolated in clinically resistant *A. fumigatus* infections have a duplication of a region of the promoter (tandem repeat, TR34), which leads to increased Cyp51A expression, in combination with a point mutation in the Cyp51A coding region. We tested the MICs of a strain harboring this TR mutation (SPF98; *cyp51A*^TR34 L98H^ compared to its voriconazole-susceptible parent, SPF93) (22) and azole-resistant clinical isolates with Cyp51A point mutations (F15861: *cyp51A*^P216L^, F16867: *cyp51A*^E427G^) (23). We did not observe any change in MICs compared to our lab reference strains (Table 1). These results suggest that the mechanism of this compound is unique from azoles and does not demonstrate cross-resistance with commonly isolated resistance mutations in clinical strains.

Next, we explored the activity of commercially available structural analogs of **1** (Fig. 1). Though we did not identify analogs of **1** with improved activity against *A. fumigatus*, we did identify compounds with intermediate activity (**3**, MIC = 64 µg/mL) and several without activity at tested concentrations (MIC >128 µg/mL). Moreover, the range of MICs of these compounds against *C. neoformans* spans two orders of magnitude, indicating that this scaffold displays a structure-activity relationship (Fig. 1). These data suggest that relatively small changes to this scaffold can modulate activity, and further medicinal chemistry optimization of this general scaffold has the potential to generate analogs with improved activity.

### 1 is active against *A. fumigatus* biofilms

**Fig 2:**
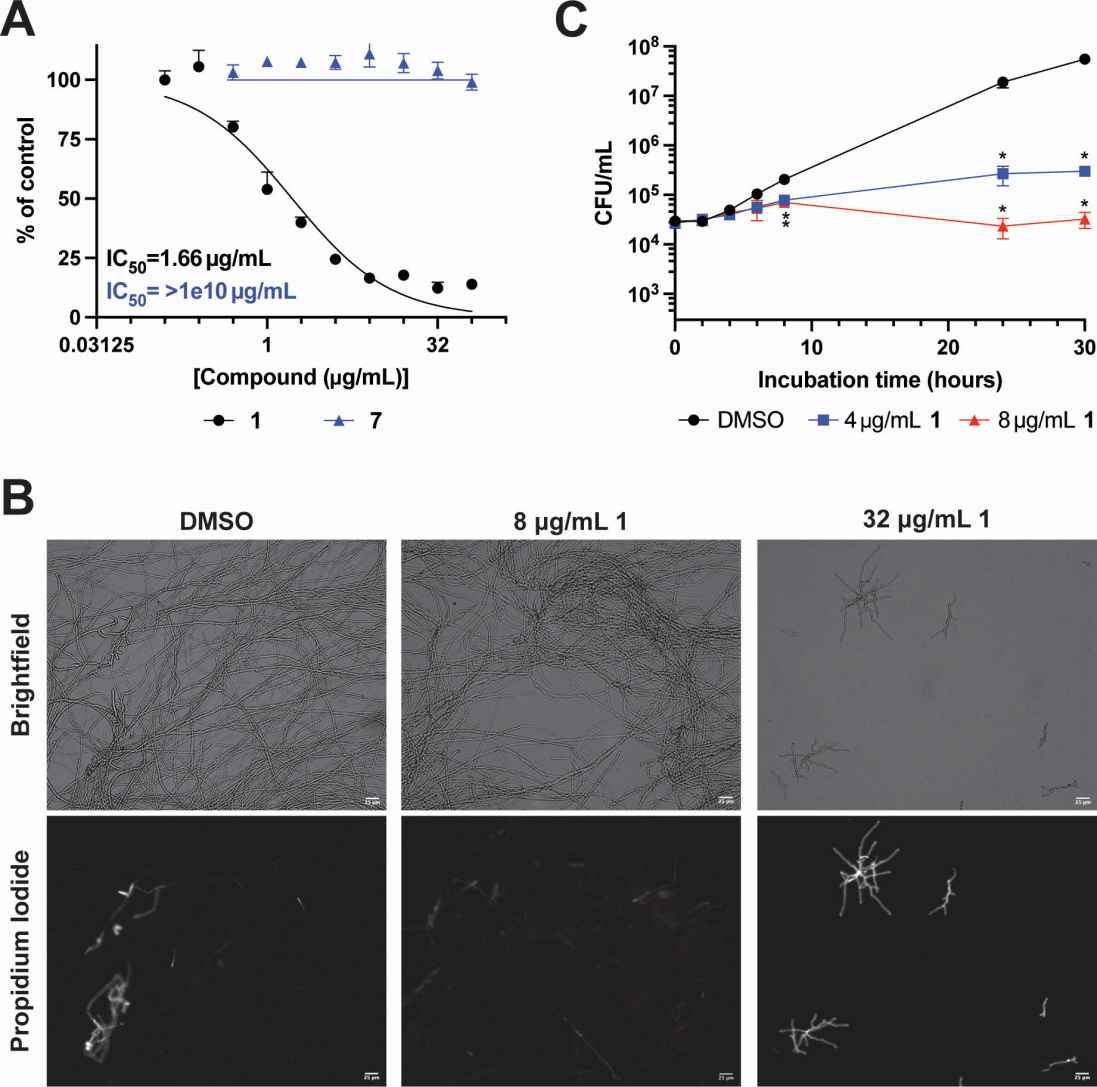
**1** is fungicidal against *A. fumigatus* hyphae. (**A**) *A. fumigatus* conidia were germinated for in the absence of drug for 16 hours, then treated with indicated doses of **1** or **7** for an additional 24 hours in the presence of the metabolic dye, resazurin. Data are represented as mean and SD of technical triplicates normalized as percent growth of vehicle-treated controls. Representative data from two independent experiments. IC_50_ values were calculated with GraphPad Prism version 10. (**B**) Propidium iodide staining of *A. fumigatus* CEA10 hyphae grown for 14 hours in the absence of drug, then treated with indicated concentrations of **1** for 24 hours. Representative images from two independent experiments performed on different days. Scale bar = 25 µm. (**C**) Viable CFUs of *C. neoformans* treated with 4 or 8 µg/mL **1** at indicated timepoints. Mean and SD of biological triplicates; representative data from two independent experiments. *P < 0.0018 by *t*-test comparison of each treatment timepoint to the respective DMSO timepoint with Benjamini-Hochberg correction for false discovery. CFU, colony-forming unit; DMSO, dimethyl sulfoxide.

Once invasive disease has been established in a patient, conidia have already undergone the morphological transition to hyphae. Therefore, it is important that mold-active antifungal drugs maintain their activity against vegetative hyphae and are not solely inhibitors of germination. To test the activity of **1** against vegetative hyphae, we allowed *A. fumigatus* conidia to germinate and initiate biofilm formation for 14 hours. The biofilms were then treated with escalating doses of **1** or the inactive compound **7** for an additional 24 hours. Using the metabolic dye resazurin as a measure of growth, we observed dose-dependent inhibition of hyphal growth by **1** with an IC_50_ of 1.66 µg/mL (Fig. 2A). As expected, the inactive analog, **7**, did not show inhibitory activity against *A. fumigatus* (Fig. 2A). Next, we stained hyphae treated with **1** with propidium iodide (PI) to determine viability following treatment. *A. fumigatus* hyphae were germinated for 14 hours then treated with 8 or 32 µg/mL **1** for 24 hours. Treatment with 8 µg/mL **1** did not show increased staining relative to vehicle-treated controls, while many cells treated with 32 µg/mL **1** stained brightly, indicating loss of membrane integrity and cell death (Fig. 2B). These results suggest that at concentrations 2× the MIC, the activity of **1** against *A. fumigatus* biofilms is fungicidal.

In contrast to *A. fumigatus*, **1** showed fungistatic activity against *C. neoformans* treated with 2× (4 µg/mL) or 4× (8 µg/mL) MIC over 30 hours. At these concentrations, viable colony-forming units (CFUs) were significantly reduced at 8, 24, and 30 hours post-treatment compared to vehicle-treated controls at each timepoint (Fig. 2C). However, we did not observe a decrease in CFUs compared to *t* = 0 of each respective treatment, indicating that cells retain viability. Consistent with this, we did not observe changes in propidium iodide of *C. neoformans* staining between vehicle-treated controls and cultures treated with **1** (Fig. S1).

Finally, we attempted to generate resistant isolates to understand the rate of resistance and potentially uncover mechanism of action. First, to determine spontaneous rates, we performed high-density plating (~2 × 10^9^ cells) of *C. neoformans* on YPD plates containing 64 µg/mL **1** (24). We were unable to isolate resistant colonies following multiple attempts with this method. Next, we employed serial passaging of both *C. neoformans* H99 and *Saccharomyces cerevisiae* BY4741 to evaluate evolved resistance upon exposure to sub-MIC concentrations. Yeast cells were grown in a 96-well format in the presence of 1/4 MIC **1**, then cultures were passaged to fresh media containing a twofold increase in drug concentration. At each passage, the inoculum was diluted ~1:100 to allow ~10 generations of growth for a total of at least 50 generations over the course of the experiment. Though both species had multiple wells with growth at drug concentrations up to 4× MIC, we did not recover stable resistance for either species after passaging strains on media without drug (data not shown). Together, these data demonstrate that spontaneous rates of resistance to **1** are low, and generating stable resistance does not occur rapidly *in vitro*.

### Chemical haploinsufficiency screen reveals signature for ER trafficking

The broad-spectrum activity and structure-activity relationship are attractive features that support the development of this scaffold. Therefore, we explored the mode and mechanism of action to determine how **1** exerts its antifungal activity on cells. To this end, we performed a chemically-induced haploinsufficiency screen using a collection of heterozygous deletion mutants composed of essential *S. cerevisiae* genes (25). Each mutant was cultured on solid plates containing vehicle or 32 µg/mL of **1**, a concentration that inhibited wild-type growth by ~50%. We identified 39 strains that were more susceptible to **1** than wild type. Next, we performed spot dilution assays with these strains to validate our screen results and score susceptibility phenotypes. Of these 39 strains, 16 had severe growth defects (+/++) and 23 had modest (+++) growth defects in the presence of **1** (Table 2; Fig. S2). Among the deletion mutants with severe growth phenotypes, we observed signatures for transcription and translation (*RPO23*, *RPL33A*, *YPL142C*, *SUI3*, *DYS1*, *RPC10*, *RPL17A*, and *RVB2*), and ER-Golgi trafficking and processing (*SEC23*, *SRP102*, and *ALG14*). We also noted several ER-Golgi-related genes (*GPI1*, *ERG7*, *RET2*, *SEC21*, and *SEC24*) among the deletion mutants with moderate growth defects.

**TABLE 2:** *S*. *cerevisiae* heterozygous mutants with growth defects in the presence of **1**^*a*^

Gene ID	Gene name	Growth^*b*^
YPR181C	SEC23	+
YPR187W	RPO26	+
YML085C	TUB1	+
YPL143W	RPL33A	+
YPL142C	N/A^*c*^	+
YPL237W	SUI3	+
YPL235W	RVB2	+
YHR068W	DYS1	++
YHR143W-A	RPC10	++
YKL154W	SRP102	++
YKL108W	SLD2	++
YKL180W	RPL17A	++
YKL189W	HYM1	++
YPR136C	Dubious_RRP9 overlap	++
YIL171W	YIL171W	++
YGL047W	ALG14	++
YDR361C	BCP1	+++
YHR107C	CDC12	+++
YDR362C	TFC6	+++
YGR190C	Dubious_HIP1 overlap	+++
YHR062C	RPP1	+++
YGR191W	HIP1	+++
YDR416W	SYF1	+++
YER012W	PRE1	+++
YGR216C	GPI1	+++
YHR072W	ERG7	+++
YDR373W	FRQ1	+++
YHR122W	CIA2	+++
YKL049C	CSE4	+++
YFR051C	RET2	+++
YNL287W	SEC21	+++
YPR035W	GLN1	+++
YMR314W	PRE5	+++
YPL131W	RPL5	+++
YIR012W	SQT1	+++
YIL144W	NDC80	+++
YIL147C	SLN1	+++
YIL150C	MCM10	+++
YIL109C	SEC24	+++
YIL104C	SHQ1	+++
YMR185W	RTP1	+++
YIL033C	BCY1	+++
YMR277W	FCP1	+++
YGL116W	CDC20	+++

^*a*^ Hits from chemical haploinsufficiency screen. Each hit was independently validated on YPD ± 32 µg/mL.

^*b*^ Growth inhibition was scored relative to wild type, (WT) as severe (+ and ++), moderate (+++), or no inhibition (++++).

^*c*^ N/A-not applicable.

**Fig 3:**
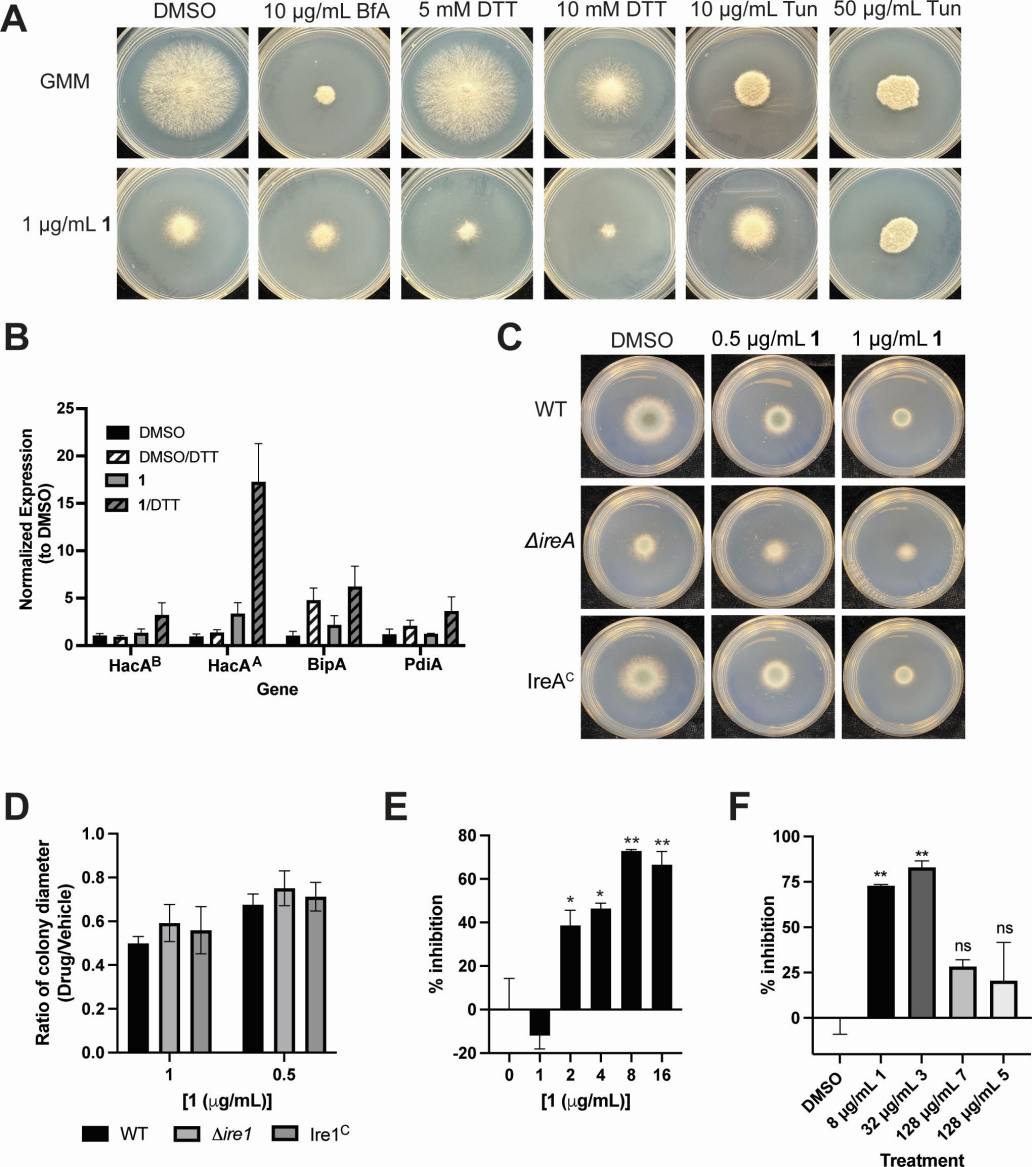
ER homeostasis and trafficking is perturbed in *A. fumigatus* treated with **1**. (**A**) CEA10 spotted on GMM containing vehicle or 1 µg/mL **1 i**n combination with ER stresses BfA, DTT, or Tun. Plates were incubated at 37°C for 72 hours. Representative images of three independent experiments with three biological replicates each. (**B**) Expression of *A. fumigatus* UPR-responsive genes in CEA10 incubated with DMSO or 16 µg/mL **1** for 2 hours at 37°C, then treated with 1 mM DTT or vehicle for an additional hour. Expression is normalized to DMSO-treated cultures. Mean and SEM of biological triplicates. (**C**) A. fumigatus CEA10 (WT), *∆ireA* and IreA^C^ were spotted on GMM with indicated concentrations of **1**. Plates were incubated for 72 hours at 30°C. The ratio of colony diameter between drug-treated and control plates is reported in panel** D**. (**D**) Mean and SEM of two independent experiments each performed in biological duplicate. (**E**) Collagenase activity of *A. fumigatus* culture supernatants treated with indicated concentrations of **1** for 24 hours in complex (10% fetal bovine serum) minimal media. Data are represented as percent inhibition compared to vehicle-treated control. Mean and SEM of biological triplicates. representative data of two independent experiments. *P < 0.015, **P < 0.002 by one-way ANOVA with Dunnett’s multiple comparisons. (**F**) collagenase activity of *A. fumigatus* culture supernatants treated with **1**, **3**, **5** or **7** at 1/2 MIC concentrations. Mean and SEM of biological triplicate (**1** and **3**) or duplicate (**5** and **7**) with technical duplicates. *P = 0.03, **P < 0.0001, compared to DMSO by one-way ANOVA with Dunnett’s multiple comparison. ANOVA, analysis of variance; BfA, brefeldin A; GMM, glucose minimal medium; ns, not significant; Tun, tunicamycin.

The strong signature for ER-Golgi trafficking prompted us to hypothesize that **1** is interfering with secretion or trafficking. To test this, we evaluated the interaction of **1** with ER stresses. We cultured *A. fumigatus* in the presence of a combination of **1** with 1,4-dithiothreitol (DTT), a reducing agent that disrupts protein folding; brefeldin A, an inhibitor of ER to Golgi trafficking; and tunicamycin, an inhibitor of protein glycosylation in the secretory pathway (Fig. 3A). Brefeldin A and tunicamycin caused modest antagonism with **1** (Fig. 3A). For tunicamycin, we observed a dose-dependent interaction where slight antagonism with **1** occurred at 10 µg/mL tunicamycin, but at 50 µg/mL tunicamycin, no interaction with **1** was observed. In contrast, DTT and **1** were synergistic. Similarly, we observed interactions between **1** and all three ER stress-inducing molecules in *C. neoformans*. Both DTT and brefeldin A were synergistic with **1**. Like *A. fumigatus*, treatment of *C. neoformans* with a combination of **1** and tunicamycin resulted in a complex interaction in which 0.125 µg/mL tunicamycin is antagonistic with **1**, while 0.25 µg/mL is additive (Fig. S3A). We interpret this dose-dependent antagonism observed for both *A. fumigatus* and *C. neoformans* at lower tunicamycin concentrations as the result of activation of an ER stress mitigation pathway that confers resistance to **1**. Together, these results suggest that **1** induces ER stress phenotypes in two distantly related fungi.

In fungi, the UPR is regulated by an ER-resident ribonuclease Ire1, which is activated in response to ER stress and splices the transcription factor Hac1 into an active form to transcribe genes required to ameliorate the accumulation of unfolded proteins (16, 26). *In A. fumigatus*, IreA splices the basal form of HacA (HacA^B^) into an activated form (HacA^A^) to transcribe UPR-responsive genes including PdiA (a protein disulfide isomerase) and BipA (an ER chaperone) (27). To assess the UPR in response to treatment with **1**, *A. fumigatus* cultures were treated with dimethyl sulfoxide (DMSO) or 16 µg/mL **1** for 2 hours, then challenged with 1 mM DTT for 1 hour. Treatment with DTT alone results in a modest increase (~1.5-fold) of the spliced form of HacA (HacA^A^) and 5- and 2-fold increases in the expression of the downstream UPR targets BipA and PdiA, respectively (Fig. 3B). Treatment with **1** alone increased HacA^A^ about threefold, though expression of the downstream target BipA was only modestly induced (2-fold) and there was no change in PdiA expression. Curiously, the combination of DTT and **1** resulted in a 3-fold increase in HacA^B^ and a 17-fold increase in HacA^A^ splicing; however, the expression of BipA and PdiA was similar to that of DTT alone (Fig. 3B). These results indicate that treatment with **1** activates the UPR, at least at the level of HacA splicing. However, UPR activation is not required for the activity of **1** because an *∆ireA* mutant, which cannot splice HacA to activate the UPR, shows the same susceptibility as wild type (Fig. 3C and D). We observed the same phenotype in the *C. neoformans ∆ire1* mutant, which did not show increased susceptibility to **1** compared to wild type (Fig. S3B).

ER trafficking in *A. fumigatus* is critical for the utilization of complex nutrient sources, which requires the secretion of hydrolytic enzymes. Therefore, to determine whether **1** impacts ER trafficking, we measured enzyme secretion during growth on a complex nutrient source using a simple assay to detect secreted collagenase (27). We cultured *A. fumigatus* in glucose media overnight, transferred mycelia to 10% fetal bovine serum (FBS)-minimal medium (MM) (10% fetal bovine serum as sole carbon and nitrogen source), then treated with vehicle or **1** for 24 hours, and collected culture supernatants. The secretion of collagenase was assessed with azocoll, a collagen substrate that is impregnated with azo dye. Culture supernatants were incubated with azocoll and release of azo dye was normalized to culture biomass. Treatment with 1 µg/mL **1** did not change collagenase secretion. However, at 2, 4, 8, and 16 µg/mL **1**, collagenase secretion was significantly inhibited compared to controls in a dose-dependent manner (Fig. 3E). Furthermore, we tested the effect of compound **3**, which has intermediate activity (MIC = 64 µg/mL), **5** and **7** , which are inactive (MIC >128 µg/mL) and observed that for active compounds **1** and **3**, the inhibition of collagenase secretion was similar when cells were treated with 1/2 MIC of each compound (Fig. 3F). For each inactive compound, we observed a modest but insignificant effect on secretion. This trend supports perturbation of ER homeostasis as the mode of action of these compounds where antifungal activity correlates with inhibition of collagenase secretion.

### Ssk2 signaling is required for adaptation to 1

**Fig 4:**
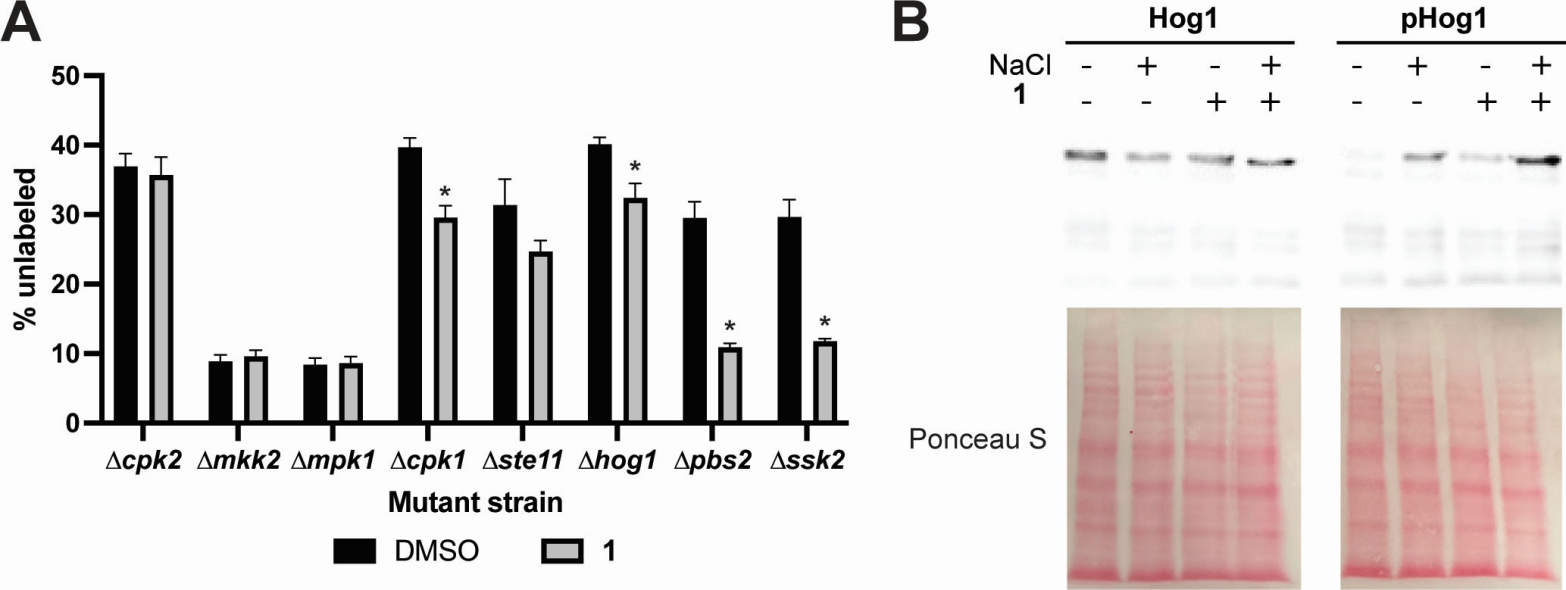
Activation of Hog1 is required for adaptation to **1**. (**A**) Competition of *C. neoformans* H99-GFP with unlabeled MAPK mutants incubated with DMSO or 1.5 µg/mL **1** for 24 hours at 37°C. Data are presented as percentage of unlabeled cells in the population. Mean and SD of biological triplicate with 100,000 cells counted per replicate. Representative of two independent experiments. *P < 0.004 by unpaired *t*-test of **1** versus DMSO for each mutant with Benjamini-Hochberg correction for multiple comparisons. (**B**) Total and phosphorylated (pHog1) Hog1 in *C. neoformans* H99 incubated with 1 µg/mL **1** for 2 hours at 30°C then treated with 1 M salt (NaCl) or vehicle for an additional 30 min. Protein extracts were run on an SDS-PAGE gel, transferred to nitrocellulose, and probed with anti-Hog1 or anti-p38 (pHog1) antibodies. Ponceau is shown as a loading control. Representative blot of two independent experiments.

Since we did not observe any effect of the loss of Ire1 on sensitivity to **1**, we screened a small, publicly available library of *C. neoformans* mitogen-activated protein kinase (MAPK) mutants to determine whether other major stress response pathways mediate responses to **1**. We performed competition experiments between a GFP-expressing wild type (H99) strain and eight unlabeled MAPK mutant strains (*∆ssk2*, *∆cpk2*, *∆mkk2*, *∆mpk1*, *∆cpk1*, *∆ste11*, *∆hog1*, and *∆pbs2*) in the presence or absence of **1** for 24 hours. We identified four strains with a significant fitness defect in the presence of **1**, including components of the high-osmolarity glycerol (HOG) pathway and *∆cpk1* (Fig. 4A). The HOG pathway mutants, *∆hog1*, *∆pbs2*, and *∆ssk2*, were reduced 20%, 60%, and 63%, respectively, when treated with **1** (Fig. 4A). Thus, we hypothesized that Hog1 is activated in response to **1** as an adaptive response. To test this, we performed Western blots probing for total and phosphorylated Hog1 in *C. neoformans* treated with vehicle or **1** for 2 hours, then challenged with salt stress (1 M NaCl) for 30 min. As expected, treatment with 1 M NaCl alone results in an increase in Hog1 phosphorylation (Fig. 4B). Treatment with **1** alone also resulted in activation of Hog1 via phosphorylation, though to a lesser extent than 1 M NaCl. The combination treatment of salt and **1** together was additive. These results demonstrate that Hog1 is phosphorylated in response to **1**, and this pathway is critical for adaptation to treatment with **1**.

### Pharmacological characterization of 1

**Fig 5:**
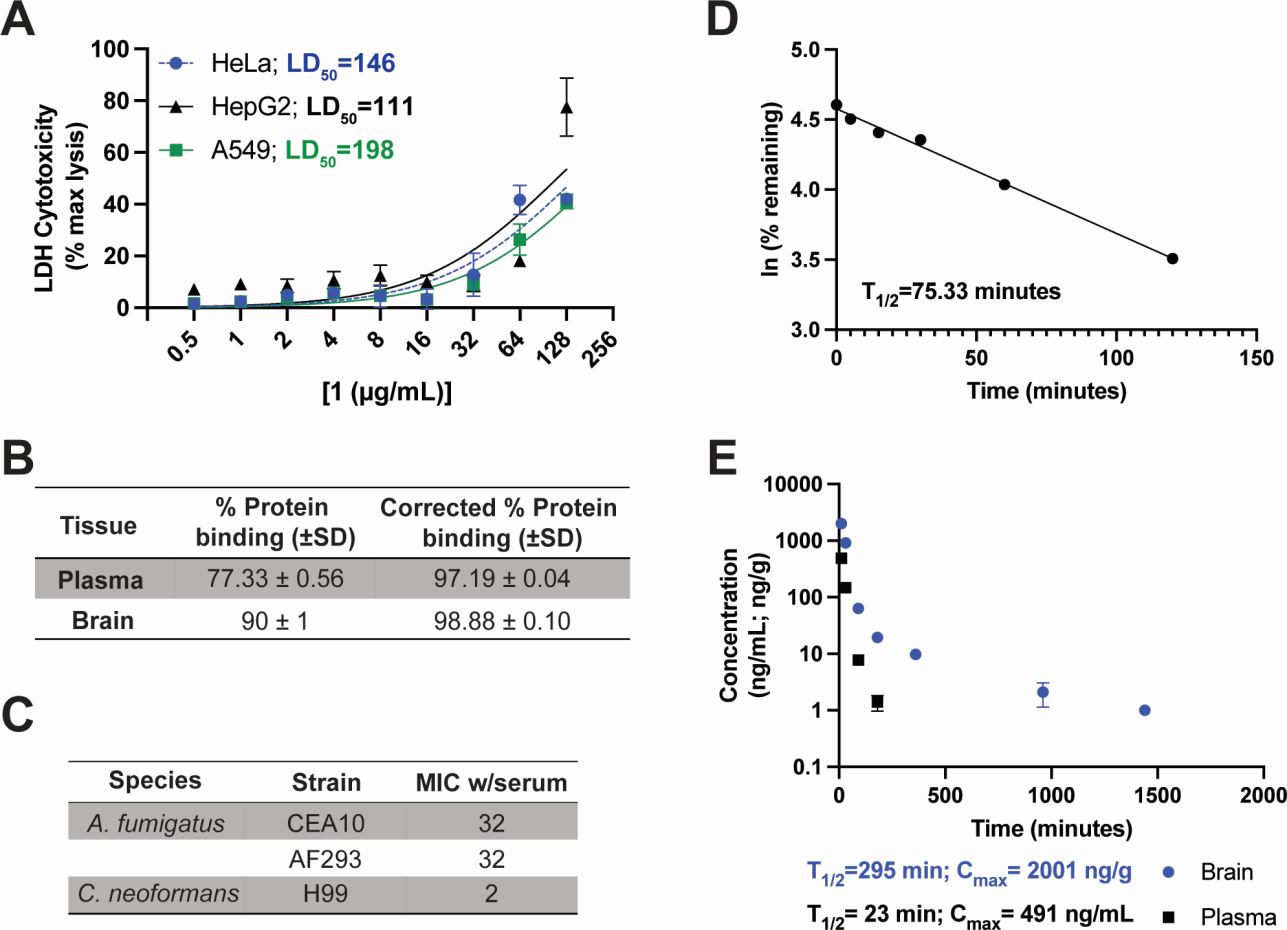
Initial toxicity and pharmacokinetic studies show promise for *in vivo* use of **1**. (**A**) Cytotoxicity of **1** against HeLa, HepG2, and A549 cells. Cells were treated with indicated concentrations of **1** for 24 hours, then cytotoxicity was measured by LDH release. Data are presented as percent lysis compared to maximum lysis control. Mean and SEM of two independent experiments performed in biological duplicate or triplicate. LD_50_ values were calculated in GraphPad Prism version 9. (**B**) Protein binding of **1** in the brain and plasma. Mean and SD of biological triplicate. (**C**) MICs (µg/mL) of **1** against *A. fumigatus* and *C. neoformans* in the presence of 10% serum. Data from two independent experiments performed on different days. (**D**) Stability of **1** in the presence of mouse microsomes. Data report a single sample per timepoint. (**E**) Serum and brain concentrations of **1** measured over 24 hours following a single 10 mg/kg intraperitoneal injection in CD1 mice. Mean and SD of *n* = 3 mice per timepoint. *T*_1/2_ and *C*_max_ are shown below the graph. *C*_max_, maximum concentration; LDH, lactate dehydrogenase; *T*_1/2_, half-life.

Finally, we evaluated the cytotoxicity and bioavailability of **1**. Importantly, we did not observe hemolysis of red blood cells treated with up to 128 µg/mL **1**, indicating this compound is not a general membrane disruptor (LD_50_ = 350 µg/mL; Fig. S4). Next, we tested the toxcitity of **1** against human cell lines including liver (HepG2), cervical (HeLa), and lung epithelial (A549) cells using lactate dehydrogenase (LDH) release as a measure of cell death. We observed LD_50_ values of 111, 146, and 198 µg/mL for HepG2, HeLa, and A549 cells, respectively (Fig. 5A). We tested the protein binding of **1** in the plasma and brain and obtained values of 97.152% and 98.88%, respectively (Fig. 5B). Despite relatively high protein binding *in vivo*, MIC testing in the presence of 10% serum showed only a moderate (2-fold) increase in MICs for both *A. fumigatus* and *C. neoformans* (Fig. 5C). Moreover, **1** was relatively stable in the presence of mouse microsomes with a half-life of 75 min (Fig. 5D).

Finally, we evaluated the bioavailability of **1** following administration of a single 10 mg/kg dose to mice intraperitoneally. We evaluated serum levels as a general measure of bioavailability and brain tissue levels to determine whether **1** can cross the blood-brain barrier. Typically, disseminated mold infections with brain involvement are associated with the highest rates of mortality, and therefore, blood-brain barrier permeability is an important consideration in antifungal development (28). In serum, **1** was short lived with a half-life of 23 min and a maximum concentration (*C*_max_) of 491 ng/mL at 10 min post-administration (Fig. 5E). However, we observed higher concentrations of **1** in the brain tissue, with *C*_max_ = 2,001 ng/g at 10 min post-administration and a half-life of 295 min (Fig. 5E). The rapid clearance of **1** from plasma *in vivo* compared to the stability in mouse microsomes suggests an alternative clearance mechanism independent of liver detoxification. Though these initial studies highlight a need for further development of this scaffold for the treatment of mold infections, the data are promising for a primary hit and provide confidence in pursuit of this scaffold as an antifungal.

## DISCUSSION

Here, we describe a novel series of pyrimidine-based compounds with broad-spectrum antifungal activity. These compounds are active against several medically important and hard-to-treat molds, including *Scedosporium*/*Lomentospora*, and *Mucor*. Since these genera often have huge intraspecies variability in drug response, we plan to expand testing of this compound to include more isolates in future studies. Preliminary toxicity studies showed moderate cytotoxicity against mammalian cells, with a therapeutic window (LD_50_:MIC with serum) of ~1:3.5 for *A. fumigatus* and ~1:27 for *C. neoformans*. Though this scaffold will require improvement for efficacy against molds *in vivo*, our initial pharmacokinetic studies are very promising and highlight the drug-like features of **1**. Despite a relatively short serum half-life, **1** penetrated the blood-brain barrier and accumulated in brain tissue with an extended half-life compared to serum. Combined with the observed structure-activity relationship of these compounds, these results demonstrate a reasonable opportunity to improve upon the anti-mold potential of these compounds with subsequent medicinal chemistry studies.

**Fig 6:**
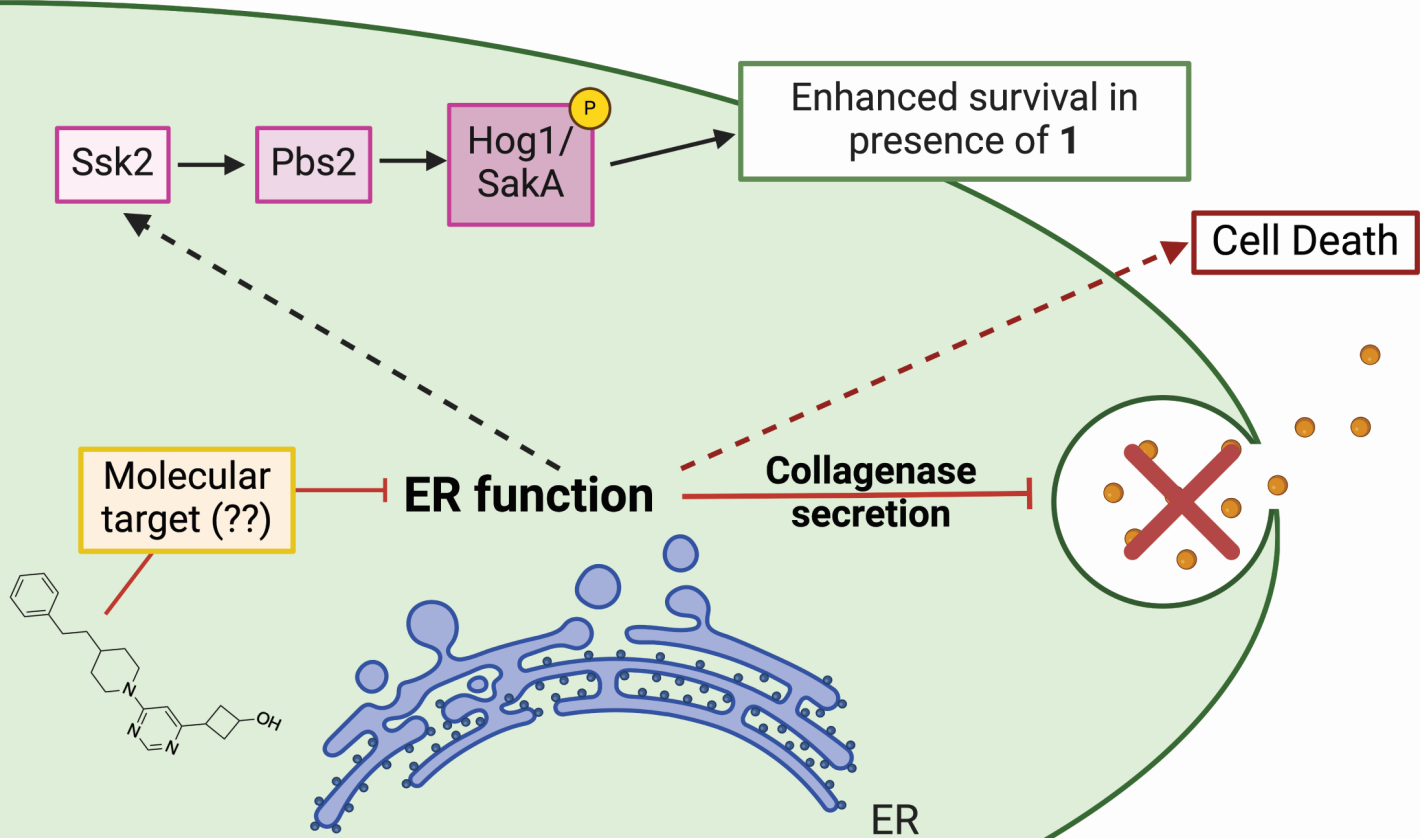
Model of activity of **1. 1** acts through an unknown molecular target to inhibit ER function and secretion of collagenase. At high concentrations, this results in cell death of *A. fumigatus* hyphae. Activation of the Ssk2-Pbs2-Hog1/SakA MAPK signaling axis, potentially in response to inhibition of ER function, results in enhanced survival in the presence of the drug.

Using genetic tools in *S. cerevisiae*, we identified several ER-related genes with increased sensitivity to **1**. We demonstrate the mode of action of **1** as perturbation of ER function through interactions with other ER stresses and the inhibition of collagenase secretion by *A. fumigatus*. However, the exact molecular target and mechanism of action of this compound remain to be determined (Fig. 6). Many of the genes we identified in our chemical haploinsufficiency screen are common hits across a broad spectrum of chemical treatments, including *TUB1* and *SEC23*, which represent the most commonly “hit” genes in chemical haploinsufficiency screens (29). Though we identified some genes that seem to respond generally to stress, we did not see any other obvious signatures in our data, so we focused on the function of genes that had the most severe phenotypes but were less common as general stress responders, including *ALG14*, *SRP102*, *DYS1*, and *HYM1*. Of these, two are directly involved in early endosome processing; *SRP102* is a subunit of the signal recognition peptide complex, which anchors ribosomes with nascent chain peptides to the translocon to facilitate the co-translation and transport of peptides directly into the ER (30, 31), and *ALG14* is a glycosyltransferase that localizes on the cytoplasmic face of the ER and catalyzes an early *N*-linked glycosylation event for protein modification (32, 33). Thus far, our evidence supports an ER target, and work is ongoing to uncover the identity of this target with these candidates at the forefront of our investigation.

Though the UPR in *A. fumigatus* is activated by **1**, IreA is dispensable for antifungal activity, suggesting that UPR activation is an indirect effect of **1** and does not drive antifungal activity. These data support a mechanism of action that is distinct from known ER inhibitors such as brefeldin A or tunicamycin, as UPR-deficient mutants are hypersusceptible to these stresses (34, 35). We did, however, identify a critical role for the MAPKKK Ssk2 and the subsequent MAPKK, Pbs2, and MAPK Hog1 in our *C. neoformans* competition experiments. Additionally, we identified the upstream histidine kinase, *SLN1*, in our haploinsufficiency screen supporting the importance of this signaling axis response to **1** (Table 2). These results are consistent with perturbations in ER homeostasis, as ER stress is mediated, in part, by the SLN1 branch of the HOG pathway in *S. cereviase* (36–38). This cross-talk requires the activity of the Ssk1/2-Pbs2-Hog1 MAPK for adaptation to ER stress, including tunicamycin and the reducing agent β-mercaptoethanol. Further, this adaptive response is independent of Ire1 and the UPR representing a distinct mechanism for maintenance of ER homeostasis (37).

We also recognize that ER stress may be an indirect effect of **1**, and the target may not be directly involved in ER function or transport. The ER responds to other cell stresses such as cell wall stress (39), iron depletion (40), and inositol depletion (41, 42). Based on our results, we do not suspect a cell wall defect in cells treated with **1**, given that we found no role for Mkk2 or Mpk1, the MAPKs that regulates the cell wall integrity pathway (CWIP), in *C. neoformans* competition assays (Fig. 4A), suggesting CWIP is dispensable for the activity of this compound. However, a role for perturbed inositol or glycerolipid signaling is an intriguing possibility, given that inositol metabolism regulates membrane homeostasis, including vesicle trafficking and cellular osmolarity. Inositol is an essential sugar precursor for the synthesis of essential glycerolipids such as phosphoinositides, sphingolipids, and glycosyl-phosphoinisitiol anchors (43). In the ER, glycerolipids are essential membrane components that help in direct assembly and activity of vesicles for transport between cellular compartments (44), which likely account for the phenotypic overlap between inositol-starved cells and secretory mutants (43). The role of sphingolipids and inositol in response to **1** is an area of active investigation.

Finally, we were unable to generate resistance mutants to **1** with two standard assays for both spontaneous and evolved resistance. The low rates of spontaneous or evolved resistance are likely due to the lack of an evolutionary path to resistance that still supports the activity of an essential cellular component. Alternatively, these results might suggest the presence of multiple molecular targets. Low rates of resistance are attractive in drug development, especially as we face the worldwide spread of azole resistance in *A. fumigatus* (23, 45). Together, this work demonstrates the potential for this novel pyrimidine-based scaffold in treating invasive fungal infections. Work is under way to identify the molecular targets, to better understand the activity of this scaffold, and, importantly, to determine whether we can improve the mold activity of this scaffold with targeted medicinal chemistry.

## MATERIALS AND METHODS

### Strains, media, and reagents

All yeast strains were maintained on YPD. All *A. fumigatus* strains were maintained on glucose minimal media (GMM) (46). All other molds were maintained on Sabouraud dextrose agar. Fungal strains are stored in 25% glycerol stocks at −80°C. *A. fumigatus* CEA10, Caf1, Caf7, *Fusarium oxysporum* DUMC Fo, and the S0− clinical series of *Aspergillus* spp. were gifts from Dr. Robert Cramer (Dartmouth College). SPF98 and SPF93 were gifts from Dr. W. Scott Moye-Rowley (University of Iowa). *C. neoformans* DUMC clinical isolate series were a gift from Dr. John Perfect (Duke University). All *Candida* spp. and H99-mNeon Green were a gift from Dr. Damian Krysan (University of Iowa). *A. fumigatus* Ku80, *∆ireA*, and IreA^C^ were a gift from Dr. David Askew (University of Cincinnati). Glucose screening medium (GSM) is a modified GMM prepared as a 2× minimal media base (1.04 g/L KCl, 1.04 g/L MgSO_4_·7H_2_O, 3.04 g/L KH_2_PO_4_ monobasic, 4.4 mg/L ZnSO_4_·7 H_2_O, 2.2 mg/L H_3_BO_3_, 1 mg/L MnCl_2_·4H_2_O, 1 mg/L FeSO_4_·7H_2_O, 0.32 mg/L CoCl_2_·5H_2_O, 0.32 mg/L CuSO_4_·5H_2_O, 0.22 mg/L (NH_4_)6Mo_7_O_24_·4H_2_O, 10 mg/L Na_4_EDTA, and 5.88 g/L L-glutamic acid; pH 6.5), combined with 20% glucose solution and water for a final concentration of 1% glucose and 1× minimal media base. Roswell Park Memorial Institute (RMPI) + 3-(N-morpholino)propanesulfonic acid (MOPS) is prepared with RPMI 1640 with L-glutamine (Gibco, Cat # 11875–093) and 0.165-M MOPS free acid (Research Products International, Cat # M92020), then pH was adjusted to 7 with NaOH and filter sterilized. Ten percent FBS-MM was prepared using 2× minimal media base, FBS (Gibco, Cat # A3160401), and water for a final concentration of 1× minimal media base and 10% FBS. DTT (Cat # D0632) was purchased from Sigma; brefeldin A (InvivoGen, Cat # inh-bfa) was purchased from VWR; tunicamycin (Cat # J62217.MA) was purchased from Thermo Scientific.

### Chemical compounds

The ChemBridge DiverSET library was obtained from ChemBridge as 10 mM DMSO stocks. Plates were diluted to 1.25 mM in 50% DMSO for screening. Compounds **2** (CID # 16131481), **3** (CID # 79226621), **4** (CID # 91509004), **5** (CID # 70521275), **6** (CID # 67783570), and **7** (CID # 28851899) were purchased from ChemBridge as powdered stocks, and compound **1** (CID # 72086400) was resynthesized from ChemBridge with greater than 95% purity and identity confirmation by liquid chromatography-tandem mass spectrometry (LC-MS/MS).

### Primary and secondary screening, hit validation

Compounds were screened using an adenylate kinase assay as previously described (18) with modifications for use in 384-well plates. Briefly, 20 µL of GSM was dispensed into each well of a white 384-well plate, followed by 0.5 µL 1.25 mM candidate drug solution, and then finally, 5 µL of conidial suspension was prepared as 5 × 10^5^ conidia/mL in 0.01% Tween-80 (Research Products International, Cat # P20390). The final concentrations of each well were 25 µM drug, 2% DMSO, and 1 × 10^5^ conidia/mL. Plates were incubated for 16 hours at 37°C, then cooled at room temperature (RT) for 2 hours before the addition of 25 µL of AK detection reagent (ToxiLight Non-Destructive Cytotoxicity BioAssay Kit; Lonza, Cat # LT07-117) per well. Plates were incubated for an additional hour at room temperature, then luminescence was recorded using a 140 ms integration time on a SpectraMax i3x multimode plate reader (Molecular Devices) with enhanced luminescence detection cartridge (Molecular Devices, Cat # 0200–7012). Data were log transformed and robust *Z* (*Z*_R_) scores were calculated for each well as previously described (47).

Primary hits were called as compounds with −3.5 > *Z*_R_ > 6,then these compounds were cherry-picked and validated. Validation testing was performed using identical conditions to our primary screen to confirm the AK phenotype. Simultaneously, each compound was tested under the same growth conditions but with the inclusion of 0.002% resazurin (Sigma, Cat # R7017) in the media to eliminate compounds that inhibit AK signal but not *A. fumigatus* growth/germination (potential inhibitors of luciferase or AK). Compounds with *Z*_R_ <−3.5 or compounds with *Z*_R_ >6 that inhibit growth by 50% or more as determined by resazurin were advanced to prioritization studies.

### Minimum inhibitory concentration testing

MICs were determined using Clinical and Laboratory Standards Institute guidelines (48, 49). All yeasts were cultured overnight in 3 mL YPD at 30°C, then washed twice in sterile phosphate-buffered saline (PBS). Twofold serial dilutions of each drug were prepared in RMPI + MOPS, then 1 × 10^3^ cells were added per well. For mold species, wells were inoculated with 1.25 × 10^3^ conidia per well. Plates were incubated at 37°C for 24 (*Candida* spp. and *S. cerevisiae*), 48 (*Aspergillus* spp., *M. circinelloides*, S. *apiospermum*, and *L. prolificans*), or 72 (*C. neoformans* and *F. oxysporum*) hours. *S. cerevisiae* BY4743 MICs were tested in YPD due to nutritional requirements of this strain. Each assay was performed in technical duplicate in at least two independent experiments performed on different days.

### *C. neoformans* time kill assay

Overnight cultures of H99 were enumerated and diluted 1 × 10^5^ CFU/mL in YPD with 4 µg/mL **1**, 8 µg/mL **1**, or DMSO. Cultures were incubated at 37°C with shaking for 30 hours with sampling at *t* = 0, 2, 4, 6, 8, 24, and 30 hours, then 10-fold serial dilutions of each sample were prepared and spotted on YPD. Viable colonies were enumerated after 48 hours at 30°C. Data are representative of two independent experiments performed in biological triplicate with technical duplicates.

### *A. fumigatus* hyphal activity, biofilm recovery, and viability staining

To determine antifungal activity against *A. fumigatus* hyphae, a 96-well plate was inoculated with 1 × 10^3^ conidia per well in 100 µL RPMI + MOPS with the edge wells filled with 200 µL sterile water to minimize edge effects. Plates were incubated at 37°C for 14 hours. A twofold dilution series of **1** or **7** was prepared in RPMI + MOPS at 2× concentration with 0.002% resazurin, then 100 µL of the drug was added to wells, and plates were incubated for an additional 24 hours. Fluorescence was measured with an excitation wavelength of 570 nm and an emission wavelength of 615 nm. Data are representative of two independent experiments performed in biological triplicate.

For PI staining of **1-**treated hyphae, 0.5 × 10^3^conidia/mL in 100 µL RPMI + MOPS were inoculated into chambers of an eight-well chamber slide and incubated at 37°C for 14 hours. 100 µL of media containing a 2× concentration of **1** or an equivalent amount of DMSO was added to each chamber, and slides were incubated for an additional 24 hours. Media were removed from chambers, replaced with PBS + 40 µM propidium iodide (Invitrogen, Cat # P3566), then slides were incubated at room temperature for 20 min in the dark. Images were acquired on a Nikon epifluorescence microscope with a Nikon DS-Fi1 camera and Nikon Elements image acquisition and analysis software. Images were processed equally with linear brightness and contrast adjustments in Photoshop, only to increase ease of viewing. Representative images of two independent experiments are shown with two biological replicates each.

### Chemical haploinsufficiency screen with *S. cerevisiae*

Chemical genetics screening was performed using the *Saccharomyces* heterozygous deletion collection (Transomic Technologies, TKY3504). Mutant strains were grown overnight in 200 µL YPD in a 96-well plate then pinned into 25 µL PBS using a 96-floating pin multiblot replicator (V&*P* Scientific Inc, Cat # 408FP6) and library copier (V&*P* Scientific Inc, Cat # 381). Cell dilutions were then pinned onto an Omni Tray (Nunc, Cat # 242811) single-well plate containing YPD with DMSO or 32 µg/mL **1** for a total of 384 spots per plate. Each plate contained at least one wild type control. Plates were incubated at 30°C and imaged every 24 hours for 4 days. Hits were called by visual evaluation. To validate hits and score growth phenotypes, serial dilutions of OD = 1.0, 0.1, 0.01, and 0.001 were prepared for each mutant from a 200 µL YPD overnight culture in a 96-well plate. Three microliters of each dilution was spotted on YPD containing DMSO or 32 µg /mL **1** and incubated for 72 hours at 30°C. The growth of each mutant on **1** was scored compared to wild-type controls as no inhibition (++++), modest (+++), or severe (+/++) growth inhibition.

### *C. neoformans* competition assay

H99-mNeon and MAPK mutants (obtained from the *C. neoformans* kinase deletion collection, FGSC) were grown overnight in YPD at 30°C then washed in PBS. Each strain was counted and diluted into 96-well plates such that each well contained 1 × 10^3^ CFU/strain with 1.5 µg/mL **1** or DMSO in 100 µL total volume. Plates were incubated at 37°C for 24 hours before analyzing on an Attune NxT Flow Cytometer with CytKick autosampler and Attune Cytometric software. Gating strategy was optimized for single cells as previously described (50). mNeonGreen positive and negative populations were identified by histogram plot with 100,000 cells counted per sample. Data represent mean and SD of biological triplicate and are representative of two independent experiments.

### Western blotting for total and phosphorylated Hog1 in *C. neoformans*

*C. neoformans* H99 overnight cultures were diluted to OD = 0.1 in YPD and incubated at 30°C for 3 hours with shaking. Cultures were split into four subcultures and treated with DMSO or 1 µg/mL **1** (two each) for 2 hours at 30°C, then water or 1 M NaCl was added to one culture of each treatment and incubated for an additional 30 min at 30°C. Cell pellets were snap frozen and stored at −80°C. Protein was extracted in extraction buffer (10 mM HEPES, pH 7.4–7.9, 1.5 mM MgCl_2_, 10 mM KCl, 1 mM DTT, 1× HALT protease and phosphatase inhibitor cocktail) by bead beading for 30 s on 60 s off with incubation on ice five times. The supernatant was removed from glass beads, and protein concentration was quantified by Bradford protein assay. Twenty micrograms of protein was loaded on a 10% SDS-PAGE gel and run at 80 V. Samples were transferred to nitrocellulose membrane for 1 hour at 100 V, then the membrane was stained with Ponceau for 15 min at RT. Membranes were blocked with 5% bovine serum albumin (BSA) in Tris-buffered saline with Tween 20 (TBST) for 1 hour at room temperature, then incubated with 1:10,000 rabbit anti-p-p38 (Phospho-p38 MAPK, # 9211; Cell Signaling) in 5% BSA/TBST or 1:5,000 custom Hog1 antibody # R02361 (GenScript) overnight at 4°C. The membranes were washed three times with TBST then incubated for 1 hour at room temperature with 1:10,000 goat anti-rabbit HRP (BioRad, Cat # STAR208P). Blots were developed with chemiluminescent substrate. Representative blots of two independent experiments were obtained.

### *A. fumigatus* UPR gene expression

We cultured 5 × 10^6^ CEA10 conidia/mL in liquid GMM for 18 hours at 37°C with shaking at 250 rpm. Cultures were treated with either DMSO or 16 µg/mL **1** for 2 hours at 37°C, 250 rpm. Then 1 mM DTT or water was added, and cultures were incubated for an additional hour. Mycelia were collected with vacuum filtration and immediately frozen with liquid nitrogen. Tissue was lyophilized then ground with glass beads with a bead beater. RNA was extracted in 1 mL of TRIazole solution (Life Technologies, Cat # 15596026), then RNA was purified with two rounds of chloroform extraction. Five micrograms of RNA was treated with TURBO DNA-free kit (Invitrogen, Cat # AM1907) according to the manufacturer’s protocol. Five hundred nanograms of DNAse-treated RNA was used for cDNA synthesis with iScript cDNA synthesis kit (BioRad, Cat # 1708891), then cDNA was diluted 1:5 with ddH_2_O. Quantitative real time PCR analysis was performed in 20 µL reactions using 2 µL of dilute cDNA per reaction with iQ SYBR Green Supermix (BioRad). Samples were run on a BioRad CFX Connect using a three-step amplification with 54°C annealing temperature and melt curve analysis. Gene expression is normalized to *tubA* and *tef1* expression. Gene expression was performed in biological triplicate. Primers used in this study are listed in Table S1. Primers to detect HacA splicing and targets were designed as previously described (51).

### ER stress interactions

To determine interactions of **1** with ER stress in *Cryptococcus neoformans*, an overnight culture of H99 was diluted to 1 × 10^6^ CFU/mL, then three 10-fold dilutions were prepared. Three-microliter spots of each cell dilution were plated in triplicate on YPD containing DMSO or 4 µg/mL **1** in combination with DTT, brefeldin A, or tunicamycin. Plates were incubated at 37°C for 72 hours. Images are representative of two independent experiments performed in biological triplicate.

For *Aspergillus fumigatus*, 5 µL of 1 × 10^4^ CEA10 conidia was spotted at the center of GMM plates containing DMSO or 1 µg/mL **1** in combination with DTT, brefeldin A, or tunicamycin. Plates were incubated at 37°C for 72 hours. Representative images of two independent experiments with three biological replicates each were obtained. For Ire1 strains, the cells were prepared as described above but were incubated at 30°C.

### Detection of secreted collagenase by azocoll

1 × 10^6^ CEA10 conidia/mL were cultured in 25 mL liquid GMM for 18 hours, then mycelia were washed with sterile ddH_2_O and transferred to 10% FBS-MM and treated with DMSO or the indicated concentration of drugs. Cultures were incubated at 37°C, 250 rpm, for an additional 24 hours, then the supernatant and biomass were collected. One milliliter of supernatant was spun at top speed for 5 min. Azocoll of <50 mesh (Sigma, Cat # 194932) was washed in buffer (50 mM Tris, pH 7.5, 1 mM NaCl, and 0.01% sodium azide) then resuspended to 5 mg/mL. Fifteen microliters of culture supernatant was added to the 2 mL azocoll solution and incubated at 37°C with shaking at 300 rpm for 3 hours in the dark. Reactions were spun at maximum speed for 3 min; 200 µL of supernatant was then transferred to a 96-well plate; and Abs 520 was measured. Biomass was lyophilized, and absorbance readings were normalized to milligram tissue. Data represent the mean and SEM of biological triplicates with technical duplicates except for compounds **5** and **7**, which are biological duplicates due to the limited availability of the drug and the required culture size for this assay.

### Mouse microsome stability

**1** (2 mM in DMSO) was incubated at 37°C with 0.5 mg/mL mouse microsomes (lot # 2110330) fraction and Phase I (NADPH regenerating system) cofactors for 0–120 min at a final concentration of 2 µM. Reactions were quenched with 0.5 mL (1:1) of methanol containing formic acid (FA) 0.2% and 100 ng/mL n-benzylbenzamide internal standard (IS; 0.1% FA and 50ng/mL IS final). Samples were vortexed for 30 s, incubated at room temperature for 10 min, and spun for 5 min at 2,200 rpm in a tabletop centrifuge. Supernatant (~0.9 mL) was then transferred to an Eppendorf tube and spun in a 4°C microfuge for 5 min at 13,200 rpm. Supernatant was transferred to an high-performance liquid chromatography plate with seal and samples then analyzed by a 4500 LC-MS/MS.

### Pharmacological characterization of 1 in mice

Twenty-one female CD-1 mice were dosed intraperitoneally with 10 mg/kg **1**. Whole blood was collected in a syringe coated with ACD. Plasma was processed from whole blood by centrifugation at 10,000 rpm for 10 min. Brain tissue was harvested and gently washed with 1× PBS to remove residual circulating blood. The tissue was weighed and snap frozen in liquid nitrogen.

#### Plasma processing

For the standards and quality controls, 98.0 and 98.8 µL of blank vendor plasma were added to a microfuge tube and spiked with 2.0 and 1.2 µL of initial standard. Standards, quality controls, and samples of 100 µL were then precipitated with 200 µL of methanol containing 0.15% formic acid and 12.5 ng/mL IS (0.1% FA and 50 ng/mL IS final). The samples were vortexed 15 s, incubated at room temperature for 10 min, and centrifuged at 13,200 rpm twice in a standard microcentrifuge. The supernatant was then analyzed by LC-MS/MS. The vendor supplied the plasma used in standards and quality controls (QCs; lot # MSE460968, ACD anti-coagulant; Bioreclamation, LLC).

#### Brain processing

Brain tissues were homogenized in 3× volume of PBS. For the standards and quality control samples, 98.0 and 98.8 µL of pooled blank brain were added to a microfuge tube and spiked with 2.0 or 1.2 µL of initial standard. Standards, quality control samples of 100 µL, were then precipitated with 200 µL of methanol containing 0.15% formic acid and 12.5 ng/mL IS (final concentrations). The samples were vortexed for 15 s, incubated at room temperature for 10 min, and centrifuged at 13,200 rpm twice in a standard microcentrifuge. The supernatant was then analyzed by LC-MS/MS.

### Protein binding analysis

Blank mouse plasma was diluted 1:10 with PBS then spiked with **1** for a final concentration of 5 µM then incubated at 37°C, 5% CO_2_, 75% relative humidity on an orbital shaker at 100 rpm for 6 hours. Stability was measured with a *T* = 0 and *T* = 6 samples. At *T* = 0, 50 µL of spiked sample was transferred to Eppendorf tubes and then matrix matched with PBS and crashed with 200 μL methanol crash (final 0.1% FA, 50 ng/mL N-benzylbenzamide). At *T* = 6 hours, *T* = 6 stability samples are matrix matched with PBS, then crashed with methanol internal standard crash. Tubes were vortexed for 10 s, incubated at room temperature for 10 min, then centrifuged at 21.1 × *g* for 5 min 2×. Two hundred microliters of the supernatant was loaded into a 96-well plate, and samples were run on Sciex 4500 LC-MS/MS.

### Mammalian cytotoxicity analysis

Hemolysis assays were performed with defibrinated sheep’s blood (Lampire, Cat # 7239001). Blood was washed three times with PBS then resuspended to ~50% hematocrit in PBS. Drugs were diluted to generate a twofold dilution series in 200 µL PBS with equal DMSO concentration across the series, then red blood cells were added for a final concentration of 2% hematocrit. Cells were incubated at RT for 2 hours in a v-bottom microtiter plate; then, plates were spun down; and the supernatant was transferred to a flat-bottomed microtiter plate for absorbance measurement at 570 nm. Drugs were tested in technical triplicate in at least two independent assays performed on different lots of blood.

HepG2 [American Type Culture Collection (ATCC), HB-8065], HeLa (ATCC, CCL-2), and A549 (ATCC, CCL-185) cells were maintained and cultured in Dulbecco’s modified Eagle medium (Gibco, Cat # 11965–092) with 10% FBS and 1% penicillin/streptomycin at 37°C with 5% CO_2_. Cells were seeded in 96-well plates at a density of 1.25 × 10^4^ cells/well and incubated overnight at 37°C with 5% CO_2_; then, media were removed and replaced with media containing a twofold drug dilution series with equal DMSO across all wells. Cells were incubated for an additional 24 hours, then the supernatant was removed and used to quantify lactate dehydrogenase release using the CyQuant LDH assay kit (Invitrogen, Cat # C20300) following the manufacturers’ directions. LDH was normalized to a maximum lysis control treated with Triton-X. HeLa and A549 cells were purchased in 2023, and the cells used for these studies were directly from fresh vials. HepG2 cells for these studies were used from a fresh, low passage (P3) vial saved directly from purchased stocks.

## SUPPLEMENTAL MATERIAL

Supplemental material spectrum.01045-24-s0001.pdf Fig. S1 to S4; Table S1.

ASM does not own the copyrights to Supplemental Material that may be linked to, or accessed through, an article. The authors have granted ASM a non-exclusive, world-wide license to publish the Supplemental Material files. Please contact the corresponding author directly for reuse.
