# Supplementary material for: A series of pyrimidine-based antifungals with anti-mold activity disrupt ER function in Aspergillus fumigatus
Source: Microbiol Spectr. 2024 Jun 25;12(8):e01045-24. doi: 10.1128/spectrum.01045-24 (PMC11302339; doi:10.1128/spectrum.01045-24)
Supplement: Supplemental material — Fig. S1 to S4; Table S1. [file spectrum.01045-24-s0001.pdf]

Supporting information

A series of pyrimidine-based antifungals with anti-mold activity disrupt ER function in *Aspergillus fumigatus*

Martin Kelty[1], Aracely Miron-Ocampo[1], Sarah Beattie[1#].

[1]Department of Pediatrics, Carver College of Medicine, University of Iowa, Iowa City, United States, 52245

[#]Corresponding author: sarah-r-beattie@uiowa.edu

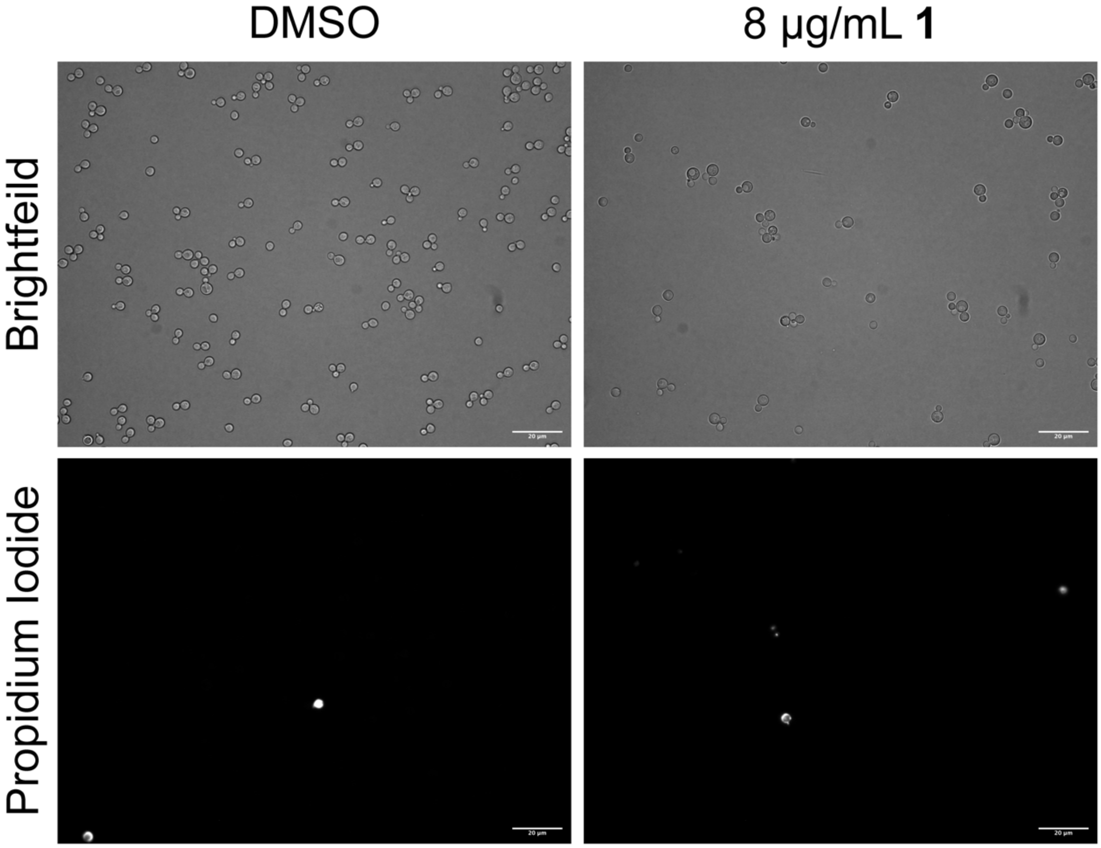

Supplemental Figure 1. 1 is fungistatic against *C. neoformans*. Brightfield and propidium iodide (PI) stained images of cells from *C. neoformans* time kill experiments at 24 hours post treatment with 8 µg/mL **1**. Cells were stained with 10 µg/mL for 10 minutes at room temperature then images were acquired on an Epifluorescence microscope. Representative images from three biological replicates with at least 100 cells imaged per sample. Scale bars = 25 µm.

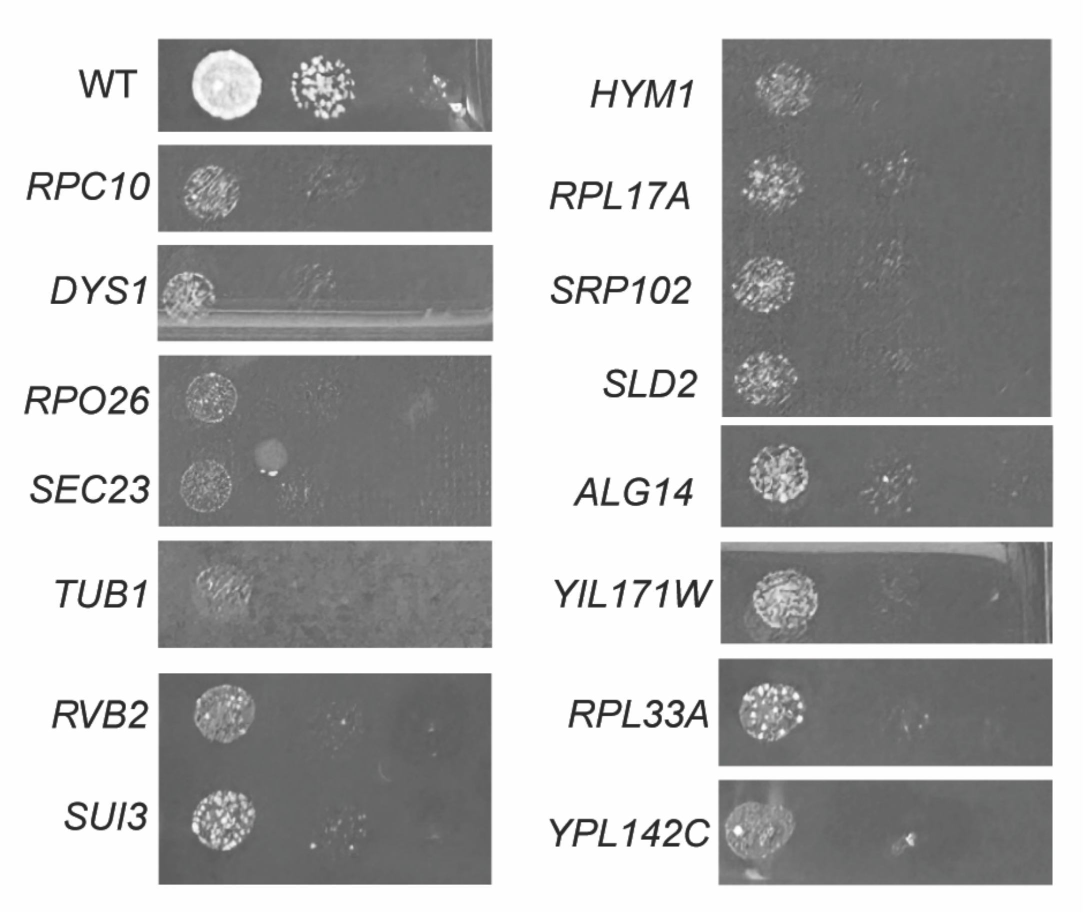

Supplemental Figure 2. Heterozygous mutants with severe growth defects on 1. Spot dilutions of *S. cerevisiae* heterozygous mutant strains spotted on YPD containing 32 µg/mL **1** incubated for 72 hours at 30°C. For all strains, growth on YPD alone was comparable to wild type.

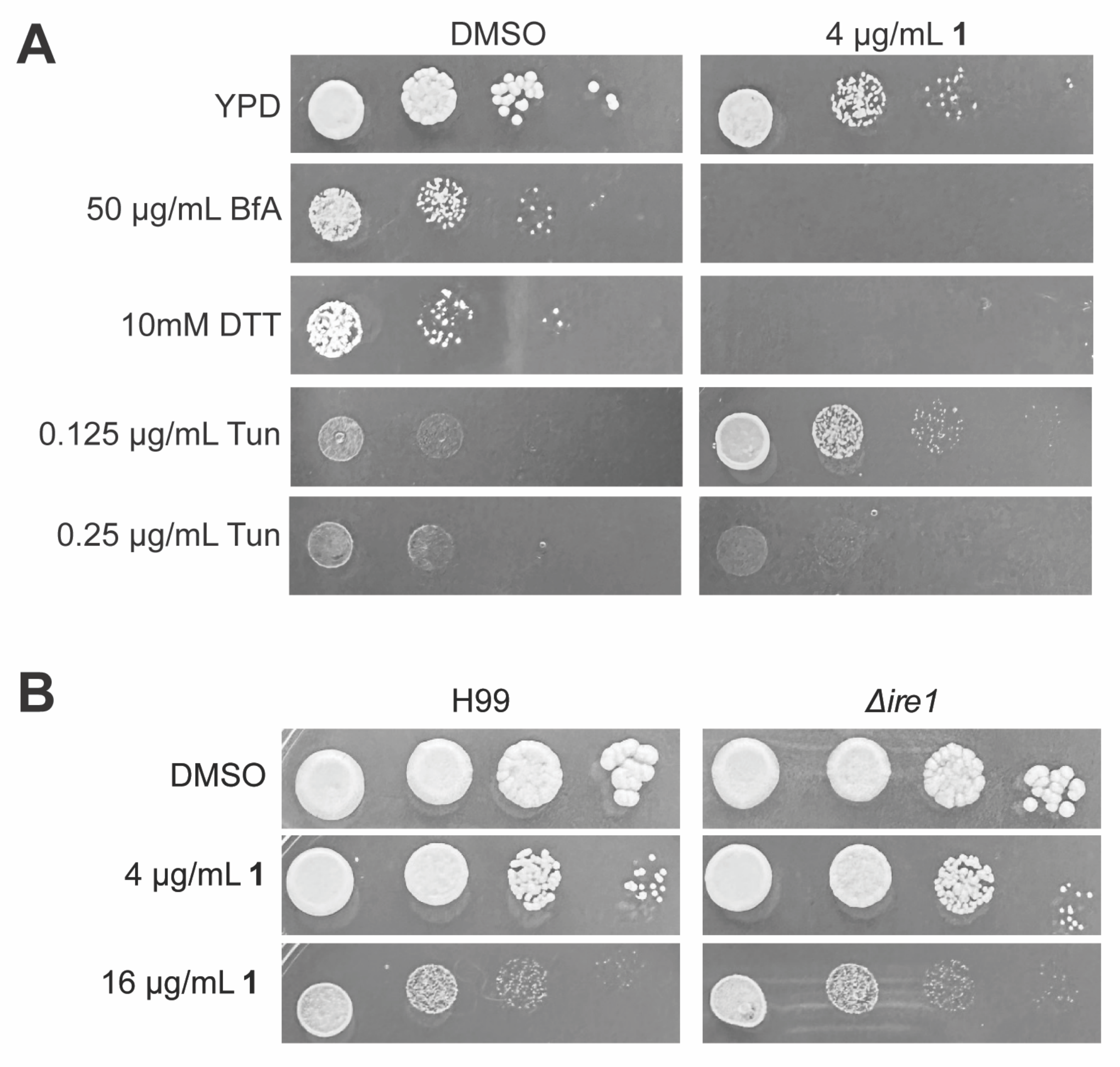

Supplemental Figure 3. 1 interacts with ER stress in *C. neoformans* but activity is not dependent on Ire1. **A)** Serial dilutions of *C. neoformans* H99 on YPD with DMSO or 4 µg/mL **1** combined with ER stresses Brefeldin A (BfA), DTT, or Tunicamycin (Tun). Plates were incubated at 37ºC for 72 hours. Representative images of two independent experiments with three biological replicates each. **B)** Serial dilutions of *C. neoformans* H99 wild type and ∆*ire1* mutant on YPD containing 4 µg/mL 1, 16 µg/mL **1** or DMSO incubated at 30ºC for 72 hours. Representative images of two independent experiments with three biological replicates each.

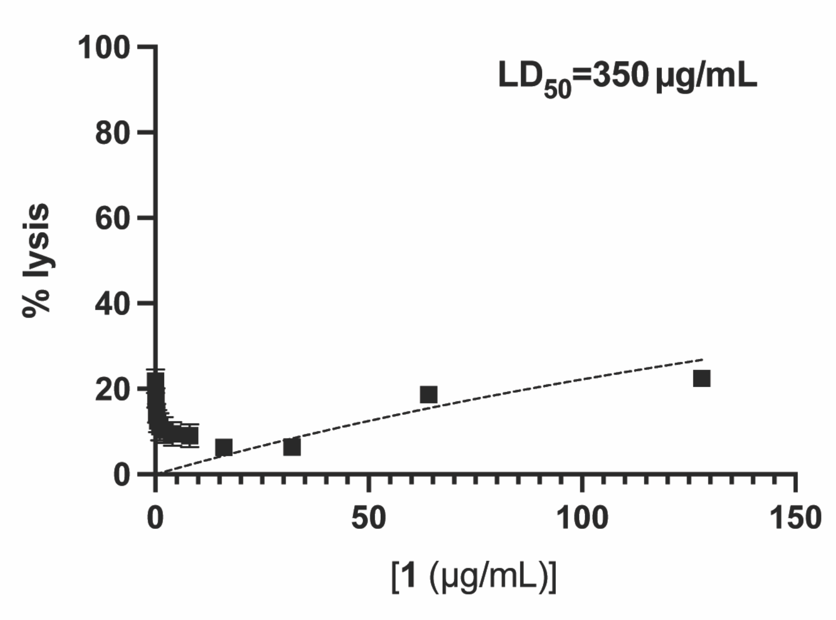

Supplemental Figure 4. 1 is not hemolytic. Commercial sheep red blood cells were diluted to ~2% hematocrit then incubated with indicated concentrations of **1** for 2 hours at room temperature. Lysis is presented as a percentage of max lysis control. Mean and SD of technical triplicates. Representative of two independent experiments performed on different lots of blood.

Primer	Description	Sequence
**SP169**	**AfTubA Fw**	TTCCCAACAACATCCAGACC
**SP170**	**AfTubA Rev**	CGACGGAACATAGCAGTGAA
**SP171**	**AfTef1 Fw**	CGACGGAACATAGCAGTGAA
**SP172**	**AfTef1 Rev**	GAACGTACAGCAACAGTCTGG
**SP266**	**HacA$^{B/A}$ Fw**	TTGTTCAAGCAAGAAGGTGATG
**SP267**	**HacA^{B} Rev**	GTCGCACAACACCGCTG
**SP268**	**AfHacA^{A} Rev**	ACTGACACTGCAGGATGTTGTG
**SP269**	**AfBipA Fw**	TGATGAAGAGCGTCTGGTTG
**SP270**	**AfBipA Rev**	TCTGGACATCCTTGTCATCG
**SP271**	**AfPdiA Fw**	TCAAGGTCGATTGCACTGAG
**SP272**	**AfPdiA Rev**	GGAGGCAAAGTAACCGATGA

Table S1. Primers used in this study.
